# Prevention, testing, and treatment interventions for hepatitis B and C in refugee populations: results of a scoping review

**DOI:** 10.1186/s12879-023-08861-1

**Published:** 2023-12-09

**Authors:** Ankeeta Saseetharran, Lindsey Hiebert, Neil Gupta, Françoise Nyirahabihirwe, Innocent Kamali, John W. Ward

**Affiliations:** 1 Coalition for Global Hepatitis Elimination, 330 W Ponce de Leon Ave, Decatur, GA 30030 USA; 2 Partners in Health/Inshuti Mu Buzima, Rwinkwavu, Rwanda

**Keywords:** Hepatitis B, Hepatitis C, Refugee, Testing, Treatment, Vaccination, Education, Linkage to care

## Abstract

**Background and aims:**

Refugees are at higher risk for hepatitis B (HBV) and hepatitis C (HCV), but often face unique healthcare barriers to vaccination, testing, and treatment. This scoping review aimed to identify and characterize HBV and HCV prevention and care services serving refugee populations globally.

**Methods:**

A literature search was conducted on Embase, Cochrane, and PubMed databases. Research studies published in English between January 2010 to July 2022 describing an HBV or HCV prevention, testing, or treatment intervention for refugees were included.

**Results:**

There were a total of 69 articles reporting viral hepatitis prevalence, implementation of services, or economic modelling. Of the 38 implementation studies, 14 were stand-alone HBV and/or HCV interventions, while 24 studies included HBV and/or HCV in an intervention targeting multiple infectious diseases and/or parasitic infections. Interventions commonly included a testing (*n* = 30) or referral (*n* = 24) component. Frequently reported features to promote program accessibility included bilingual services (*n* = 25), community partnerships (*n* = 21), and multidisciplinary staff members (*n* = 18), such as cultural and/or linguistic mediators, community health workers, community health leaders, lay health workers, local health staff, members of the refugee community, and social workers. The most commonly reported challenge was the transience of refugees (*n* = 5). Twenty studies noted funding sources, of which twelve reported governmental funding (not including national health insurance) and eight reported that refugees received national health insurance.

**Conclusions:**

This is the first scoping review to characterize the types of hepatitis prevention, screening, and treatment interventions serving refugee populations globally. Published experiences of HBV and HCV services for refugee populations remain limited. Additional efforts are needed to disseminate models of hepatitis interventions for refugees to ensure access to care for this key population. To achieve hepatitis elimination globally, best practices must be identified and shared to expand access to hepatitis services for refugee populations.

**Supplementary Information:**

The online version contains supplementary material available at 10.1186/s12879-023-08861-1.

## Background

Ensuring that all populations have equitable access to hepatitis B (HBV) and hepatitis C (HCV) prevention, testing, and treatment services is critical to achieving HBV and HCV elimination. Refugee, asylum seekers, and internally displaced persons have been shown to have a high prevalence of HBV and HCV in many settings. High HBV seroprevalence has been reported in at least eight settings: Syrian refugees in Turkey (1–5%); Myanmar refugees along the Thai-Myanmar border and in Thailand (6–10%); Afghan refugees and asylum seekers in Pakistan, Turkey, and Iran (8–61%); refugees in Gambella, Ethiopia (7%); refugees in Athens, Greece (15%); refugees at the Muzaffarabad refugee camp in Pakistan (7%); Burundian refugees at the Mahama camp in Rwanda (4%); and Rohingya refugees in Bangladesh (4%) [1–6]. Similarly, high HCV seroprevalence has been reported among refugees in Gambella, Ethiopia (2%), among refugees in Athens, Greece (2%), among refugees residing in the Muzaffarabad refugee camp in Pakistan (18%), and Rohingya refugees in Bangladesh (11%) [2–6]. Refugees residing in Australia, Canada, New Zealand, the United States, and 18 countries in Europe who are originally from countries with intermediate to high HBV and HCV endemicity are at high-risk for HBV and HCV [7, 8].

Despite the high burden of HBV and HCV, refugees are less likely to be screened and treated for HBV and HCV, and face limited to no access to routine health care compared to the general population [9]. They may experience disrupted health services, have a low awareness of hepatitis, experience stigma and fears around hepatitis, and face high costs for screening and treatment [9, 10]. The barriers to care that they face put them at higher risk of late diagnosis and advanced HBV- and HCV- related liver disease [10]. Previous scoping and literature review articles have identified a multitude of barriers around healthcare accessibility for refugees, including: language, health literacy, poverty, transience, dissatisfaction with healthcare services, poor continuity of care, perceived discrimination, culturally inappropriate care, and limited knowledge of healthcare infrastructure [11, 12]. However, previous reviews have not identified key characteristics and strategies for improving coverage of hepatitis services among refugee populations. This scoping review aimed to identify and characterize published experiences of HBV and HCV prevention, testing, and treatment interventions serving refugee populations to date in order to inform the development of improved policy and service delivery.

## Methods

### Information sources and search strategy

The literature search was conducted from August to October 2022 on Cochrane, Embase, and PubMed online databases for articles published in English. The main search strategy included relevant keywords for HBV, HCV, education, testing, referral to care, treatment, harm reduction, and refugees (Supplementary Table 1). Retrieved records were organized in Endnote.

### Eligibility criteria and study selection

**Fig. 1:**
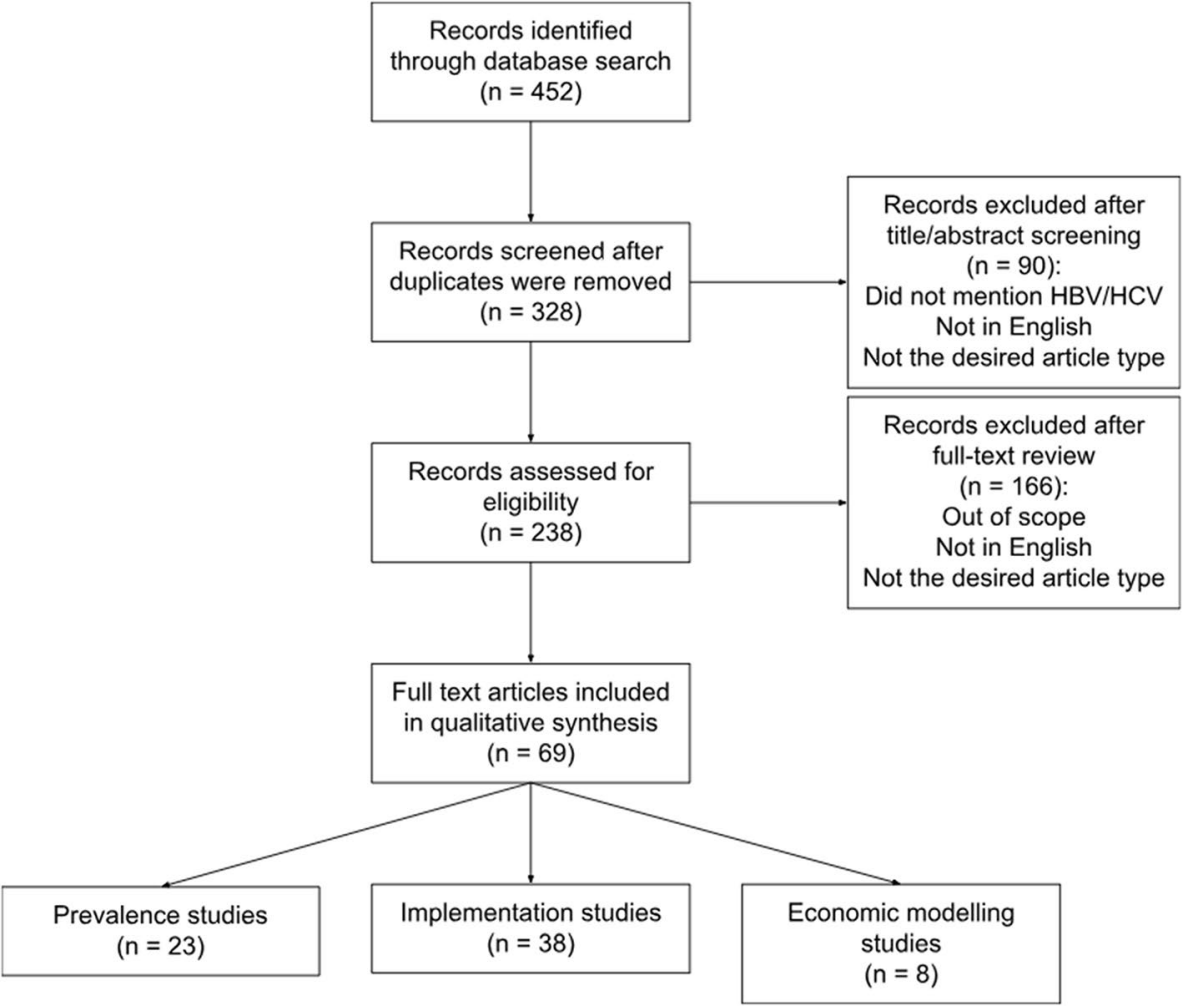
Flow diagram from identification to inclusion. Legend: The PRISMA flow diagram shows the systematic process from the initial search to the final inclusion of articles

An independent reviewer conducted the title/abstract screening and the full text review. The inclusion timeframe was a publication date between January 2010 to July 2022. Articles were eligible for inclusion after the title and abstract screening if they mentioned HBV or HCV and if the study population included refugees, asylum seekers, or internally displaced persons. Articles were included after the full text review if they described an HBV or HCV prevention, screening, or treatment intervention for refugees, asylum seekers, or internally displaced persons, which required a reference to cost, equipment, recruitment or outreach, program evaluation, or staffing. During both the title and abstract, and full text screening stages, records were excluded if they were not in English for comprehension purposes. Records were also excluded if they were a presentation, stand-alone abstract, recommendation, guideline, study protocol, case report, editorial, letter, or commentary in order to exclude articles with insufficient data reporting (Fig. 1).

As defined by the United Nations High Commissioner for Refugees, refugees are individuals who have been forced to flee and find safety in another country [13]. Asylum seekers are individuals whose requests for sanctuary in another country have not been processed [14]. Internally displaced persons are individuals who have been forced to flee their home but do not cross into another country [15]. Articles were considered if they explicitly used the term “refugee,” “asylum seeker,” or “internally displaced person” to describe any of their study participants.

Studies were grouped into three categories based on their primary aim: program implementation reporting, prevalence estimation, or economic modeling. Implementation studies were defined as studies that described the operational details of delivering prevention, testing, or treatment services for refugee populations. Prevalence studies were defined as studies that discussed systematic screening efforts undertaken to determine the disease burden in a specific population. Economic modeling studies were defined as studies that described the use of mathematical modeling to estimate the cost or cost-effectiveness of potential interventions among refugees.

### Data items and extraction

The following characteristics were recorded during the full text review from all three groups of studies unless otherwise indicated: country of study, disease targeted (HBV, HCV, both, or integrated), years of intervention, included population, number of individuals reached, intervention setting, and host organization. Additional data was recorded for each type of study as appropriate. From prevalence studies, the prevalence of HBV markers, hepatitis C antibody (anti-HCV), and hepatitis C ribonucleic acid (HCV RNA) were recorded as percents. HBV markers included hepatitis B surface antigen (HBsAg), total antibody to hepatitis B core antigen (anti-HBc), hepatitis B deoxyribonucleic acid (HBV DNA), and hepatitis B surface antibody (HBsAb). When necessary, percentages were calculated with the numerical data reported for individuals testing positive and total number of individuals tested. From economic modelling studies, key economic analysis takeaways were extracted.

The following information was recorded for implementation studies: intervention type (either education, harm reduction, vaccination, testing, referral to care, and treatment); partner organization(s); staffing; outreach strategy; point-of-care testing; reflex testing; costs; funding source; program outcomes (ie. screening coverage, vaccination coverage, treatment uptake); program accessibility features related to healthcare barriers (language, health literacy, poverty, transience, satisfaction with healthcare, continuity of care, perceived discrimination, culturally appropriate care, and knowledge of healthcare infrastructure); and intervention challenges as described by the study. Education included pre-test counseling, treatment counseling, and/or general hepatitis education. Testing included testing for anti-HCV, any HBV marker, and/or viral load. Reflex testing is when a single blood specimen sample is used for antibody testing and subsequent molecular confirmation if the initial antibody test was positive [16]. Harm reduction included interventions to minimize the negative impacts of drug use, such as education on safer drug use, needle and syringe programs, and opioid therapy [17]. Program outcome data were recorded as percentages with numerator and denominator data whenever possible. If one of these three values were not provided, they were calculated with the other two data points provided.

### Evaluation of individuals studies and synthesis of results

Based on the extracted data, a descriptive analysis was conducted on each group of studies. For prevalence studies, study locations and prevalence estimates were summarized. For implementation studies, intervention characteristics (i.e., location, disease targeted, type, and cost), as well as common program accessibility features and challenges were synthesized. For economic modelling studies, the key economic results were summarized.

## Results

A total of 328 unique reports were identified. After a full text review, 69 studies were from 26 countries were included in the scoping review; all 6 World Health Organization (WHO) regions were represented. Of the 69 studies, a total of 38 were implementation studies, 23 were prevalence studies, and eight were economic modeling studies (Supplementary Table 2).

### Prevalence studies

The 23 prevalence studies are from 16 countries, including: Australia (*n* = 1), Bangladesh (*n* = 1), Cameroon (*n* = 1), Denmark (*n* = 1), Ethiopia (*n* = 1), Germany (*n* = 2), Greece (*n* = 1), India (*n* = 1), Italy (*n* = 4), Nigeria (*n* = 1), Pakistan (*n* = 4), Rwanda (*n* = 1), Spain (*n* = 1), Switzerland (*n* = 1), Turkey (*n* = 1), and the United States (*n* = 1). These prevalence studies were conducted at clinics or hospitals (*n* = 8), medical camps (*n* = 1), reception centers (*n* = 2), refugee camps (*n* = 7), and refugee centers (*n* = 1) (Supplementary Table 3). Four studies assessed the prevalence of HBV only and nineteen studies assessed the prevalence of both HBV and HCV. No studies assessed HCV prevalence alone. Studies generally tested and reported HBsAg, anti-HBc, HBV DNA, and/or HBsAb for HBV. For HCV, studies tested and reported anti-HCV and/or HCV RNA. The prevalence of HBsAg ranged between 0–23% as reported by 22 studies and the prevalence of anti-HCV ranged between 0–20% as reported by 19 studies (Supplementary Table 4).

### Implementation studies

The 38 implementation studies were conducted in 11 countries: Australia (*n* = 4), Finland (*n* = 1), France (*n* = 2), Germany (*n* = 2), Greece (n = 1), Italy (*n* = 10), Norway (*n* = 1), Rwanda (*n* = 1), Thailand (*n* = 3), United Kingdom (*n* = 1), and the United States (*n* = 12). Interventions were implemented in: clinics or hospitals (*n* = 20); reception, immigration, or asylum seekers centers (*n* = 3); refugee shelters or accommodations (*n* = 3); and refugee camps (*n* = 2). All intervention regions and settings are shown in Table 1. Twenty-four studies targeted multiple infectious diseases and parasitic infections, including HBV and/or HCV. One study targeted HBV and HCV. Eleven studies targeted HBV only, and two studies targeted HCV only. The most common interventions or intervention packages were stand-alone testing (*n* = 5) and testing and referral to care combined (*n* = 5). No studies included a harm reduction component to reduce the risk of HBV or HCV transmission among persons who inject drugs (Table 2).

**Table 1:** Implementation study location and setting (*n* = 38)

**A. Implementation study location**	
Country	Frequency (%)
Australia	4 (11)
France	2 (5)
Finland	1 (3)
Germany	2 (5)
Greece	1 (3)
Italy	10 (26)
Norway	1 (3)
Rwanda	1 (3)
Thailand	3 (8)
United Kingdom	1 (3)
United States	12 (32)
**B. Implementation study setting**	
Setting	Frequency (%)
Clinical or hospital	20 (53)
Clinical sites and mobile teams	1 (3)
Community center	1 (3)
Multiple settings	6 (16)
Reception, immigration, or asylum seekers centers	3 (8)
Refugee camps	2 (5)
Refugee shelter or accommodations	3 (8)
Research institute	1 (3)
No data on study setting^a^	1 (3)

^a^All studies reported data on study location. However, not all studies reported data on study setting

**Table 2:** Intervention type and details (*n* = 38)

**A. Disease included in intervention**	**Frequency (%)**
Includes HBV	22 (58)
Vertical HBV intervention	11 (29)
Integrated with other disease	11 (29)
Includes HCV	3 (8)
Vertical HCV intervention	2 (5)
Integrated with other diseases	1 (3)
Includes HBV and HCV	13 (34)
Vertical HBV and HCV intervention	1 (3)
Integrated with other diseases	12 (32)
**B. Summary of intervention types**	**Frequency (%)**
Intervention type	
Education	19 (50)
Testing	30 (79)
Referral to care	24 (63)
Treatment	11 (29)
Vaccination	12 (32)
**C. Summary of stand-alone interventions vs. intervention packages**	**Frequency (%)**
One intervention type	
Education	3 (8)
Testing	5 (13)
Vaccination	3 (8)
Multiple intervention type	
Testing, Referral to care	5 (13)
Testing, Referral to care, Treatment, Vaccination	4 (11)
Education, Testing, Referral to care	4 (11)
Education, Testing	3 (8)

Of the 38 implementation studies, 30 studies included a testing component. Seven studies reported using HBV and/or HCV point-of-care antibody tests, fifteen studies did not use point-of-care tests, and eight studies did not report adequate data to determine point-of-care testing utilization. Eleven studies reported using reflex testing. One study included multiple sites of which some sites conducted reflex testing. Fourteen studies did not conduct reflex testing. Four studies did not provide adequate data to determine if reflex testing was conducted (Supplementary Table 5).

The most common approach for recruitment was requesting partner organizations to refer individuals (*n* = 10). Additionally, five studies involved community health workers, cultural mediators, community leaders, religious leaders, or social workers in the recruitment process (*n* = 5). Recruitment was most commonly conducted at clinic or hospital visits (*n* = 8) and at refugee accommodations or residential areas (*n* = 5). Eleven studies did not provide any information on their recruitment strategies (Table 3).

**Table 3:** Recruitment approaches reported by implementation studies (*n* = 38)

Recruitment approaches	Frequency (%)^a^
Recruitment strategies	
Door-to-door visits	2 (5)
Flyers or posters	3 (8)
Phone calls	2 (5)
Radio and television	2 (5)
Referred by migrant center, health care professionals, asylum lawyers, community organizations, resettlement agencies, etc	10 (26)
Supported by CHWs, cultural mediators, community leaders, religious leaders, or social workers	5 (13)
Word of mouth	4 (11)
Recruitment locations	
Clinic or hospital visits	8 (21)
Community events and locations	2 (5)
Faith based locations	3 (8)
Grocery stores, shops, and businesses	3 (8)
Refugee accommodations, apartment complexes, camps	5 (13)
Resettlement agencies	1 (3)
Restaurants	1 (3)
No data on recruitment strategies or locations	11 (29)

^a^Studies varied in how many strategies or locations they reported, so the percents do not total to 100%

Commonly reported features promoting accessibility included: bilingual care, education, services, and test notifications (*n* = 25); partnerships with community organizations, hospitals, and other stakeholders (*n* = 21); and multidisciplinary team members (i.e., cultural and/or linguistic mediators, community health workers, community health leaders, lay health workers, local health staff, members of the refugee community, and social workers) (*n* = 18). Additionally, transportation assistance was incorporated in five interventions, such as transportation vouchers, local agreements to improve transportation access, and arranging free transportation for patients through the patient’s clinic, patient’s medical plan, or the intervention’s taxi fund. Moreover, four interventions commented on how the use of photos and diagrams helped address language barriers. Two studies did not report any program accessibility features. See Table 4 for a complete list of reported program accessibility features.

**Table 4:** Program accessibility features reported by implementation studies (*n* = 38)

Program accessibility features reported by implementation studies	Frequency (%)
Assistance with healthcare navigation	4 (11)
Bilingual care, education, services, and test result notifications	25 (66)
Provided by cultural or linguistic mediators	4 (11)
Provided by community health workers	3 (8)
Provided by interpreters or translators	11 (29)
Provided by volunteers	1 (3)
Provided by members of the refugee community	2 (5)
Provided by social workers	1 (3)
On-demand healthcare availability, no appointments needed	1 (3)
Clinic is open 24/7	1 (3)
Cultural considerations were taken (ie. culture based training provided to staff, culturally themed educational slides and activities, ethnic food provided to educational workshop participants, etc.)	9 (24)
HBV care integrated with antenatal care	1 (3)
HBV disease registry for managing care	2 (5)
In-house PCR systems originally used during soldier screenings were utilized for refugees	1 (3)
Partnerships with community organizations, refugee centers, laboratories, hospitals, and/or other stakeholders	21 (55)
Phone outreach	5 (13)
Physicians and/or staff traveled to refugees' residences (ie. mobile vaccine teams)	3 (8)
Remote or electronic data entry and/or data transfer	5 (13)
Services provided free of charge to patients	7 (18)
Services provided regardless of ability to pay	1 (3)
Staff includes cultural mediators, linguistic mediators, community health workers, community health leaders, lay health workers, local health staff, members of the refugee community, and social workers	18 (47)
Timely care provision	4 (11)
Transportation assistance	5 (13)
Use of diagrams, illustrations, or photographs for medical terms and to overcome language barriers	4 (11)
No data	2 (5)

Common challenges across all intervention types included the mobility of refugee populations (*n* = 5), language or communication barriers (*n* = 4), and equipment, supply, or medicine limitations (*n* = 4). Loss to follow up was the leading challenge among interventions with a referral to care component (*n* = 4). Difficulty monitoring vaccination status was the leading challenge among interventions with a vaccination component that reported on challenges (*n* = 3). Eight studies did not report any challenges faced during program implementation. All commonly reported challenges are shown in Table 5.

**Table 5:** Common challenges reported by implementation studies (*n* = 38)

Common challenges	Frequency (%)
Delays (ie. in starting vaccination, screening, etc.)	2 (5)
Difficulty monitoring vaccination status	3 (8)
Difficulty completing vaccination series	2 (5)
Equipment, supply, and medicine limitations	4 (11)
Lack of knowledge and skill among staff members	2 (5)
Lack of medical knowledge among refugees	3 (8)
Language or communication barriers	4 (11)
Linkage to care or treatment refusal	2 (5)
Loss to follow up after screening	4 (11)
Refugees moved out of the area	5 (13)
Staffing inefficiencies	2 (5)
Stigma and fear in refugee communities	1 (3)
No data	8 (21)

Overall, 27 of the 38 implementation studies reported details about program impact, including screening coverage, vaccine uptake, linkage to care rates, and/or treatment outcomes (Supplementary Table 6). Fifteen articles reported screening coverage. Of the 14 articles that reported HBV screening coverage, coverage ranged from 26–96% for HBV and seven reported a screening uptake of over 75%. Of the seven articles that reported HCV screening coverage, coverage ranged from 25–95% and five reported a screening uptake of over 75%. Eight articles reported vaccine coverage: four articles reported vaccine coverage for all three doses, two articles reported vaccine coverage for two doses, and two articles reported vaccine coverage for only the first dose. Six of these interventions provided vaccinations to both adults and children, and two provided vaccinations to children only. There were no reports of hepatitis B birth dose implementation or coverage data. Vaccine coverage varied widely from 0.5–99% for the first dose, 0.2–25% for two doses, and 0.03–92% for three doses. Ten articles reported referral to care outcomes: all ten articles assessed HBV linkage to care, while only one assessed HCV linkage to care. Linkage to care rates varied between 11%-94% for HBV. Two articles reported treatment outcome information. Of the 14 individuals who received HBV treatment, ten obtained a favorable response. Of the eight individuals who received HCV treatment, six were cured.

Details about funding sources were provided by 20 of the 38 studies. Twelve studies reported receiving dedicated governmental funding beyond leveraging national health insurance coverage, eight studies reported that refugees were included in the national health insurance scheme, and three studies reported receiving in-kind commodity donations (Supplementary Table 7).

### Economic modelling studies

Eight studies assessed the cost and cost-effectiveness of HBV interventions for refugees (Supplementary Table 8). Of these eight studies, six were from high-income countries: Australia, Canada, Germany, and United States. Available economic studies utilized a range of methodological approaches and found varying results across various settings. Subramaniam et al. found that without HBV treatment for refugees, there would be increased costs to the Australian healthcare system due to caring for refugees living with unmanaged HBV [18]. Rossi et al. found that in Canada, screening and treatment were more cost-effective than any intervention that included vaccination [19]. Bozorgmehr et al. found that costs associated with HBV screening were the highest out of all of the infectious diseases being screened for among refugees in Germany, and costs were higher with private health insurance versus statutory health insurance [20]. Two studies from the United States, Chahal et al. and Jazwa et al., found that bundling screening, vaccination, and treatment together for refugees was cost-effective [21, 22]. Adachi et al. found that a clinic in the United States broke even or had a slightly positive cost-revenue structure when they provided hepatitis B vaccines to refugees of all ages as part of the standard package [23]. Two studies were from refugee camps in South Sudan and the African region, Gargano et al. and Reardon et al., and supported the cost-effectiveness of hepatitis B vaccination, either in conjunction with routine immunization or pneumonia immunization [24, 25].

## Discussion

This scoping review was the first to systematically characterize published reports of interventions for HBV and HCV care delivery in refugee populations globally. The majority of interventions (63%) involved a general infectious and parasitic disease screening program that included HBV and/or HCV or a general vaccination program that included HBV.

Despite a high prevalence of HBsAg and anti-HCV among refugees, ranging up to 23% [26] and 20% [26], respectively, there is a glaring lack of published experiences on interventions for refugee populations in global settings. Only 55% of the 69 studies included in this scoping review were implementation studies, as opposed to prevalence or economic modelling studies. Only 11% of interventions were identified in low- or middle-income countries, which includes one study from Rwanda and three studies from Thailand. Detailed operational and program impact reporting was also lacking. Only 53% of implementation studies provided information regarding sources of funding. About 70% of implementation studies provided information on recruitment strategies, and 70% of implementation studies provided outcome information, such as screening or vaccination coverage, linkage to care rates, and treatment outcomes.

Additional and improved models for linkage to care, treatment, and vaccination for refugees are needed due to the reported challenges and inconsistency of impact reporting across programs. Treatment and vaccination interventions were least commonly implemented. Hepatitis B birth dose implementation was also not reported. Furthermore, common challenges related to linkage to care, treatment, and vaccination included loss to follow up after screening, linkage to care/treatment refusal, difficulty monitoring vaccination status, and difficulty completing vaccination series. Impact data, when available, varied widely for the above intervention types.

Community engagement was a common theme among the key accessibility features reported by studies. As an example, the HBV and HCV Screening Campaign at the Mahama Refugee Camp showed that to better organize, manage screening activities, involving the community played a key role through: (i) community representatives who scheduled specific days and screening locations for each of the villages within the refugee camp, and (ii) volunteer community health workers who conducted door-to-door visits to prepare households for screening and mobilize individuals to attend screening on their villages’ scheduled day (personal communications with Partners in Health/Inshuti Mu Buzima).

Cultural mediators, community health workers, refugee staff members, and other support staff were involved in recruiting participants, interacting with refugees during the intervention, assisting refugees in navigating the healthcare system (ie. scheduling appointments), referral to care, clinical consultations, and educating refugees on the importance of hepatitis prevention, testing, and treatment. Intervention hosts commonly partnered with community stakeholders and employed members of the community that they were serving, which was beneficial for securing program sites, recruiting participants, obtaining supplies, and other implementation logistics (ie. providing vaccinations).

National level financial support appears to be essential to hepatitis service delivery for refugees. Twelve studies reported governmental funding (excluding national health insurance) and eight studies reported that refugees were included in the national health insurance scheme. Furthermore, the economic modelling study from Germany found that HBV screening costs were higher with private health insurance versus statutory health insurance, supporting the cost-benefits of including refugees in the statutory health insurance scheme [20].

Promoting continuity of care for refugee populations as they relocate could be beneficial to ensuring successful linkage to care and vaccination completion. Five studies mentioned the refugees’ mobility as a challenge. While challenges to linkage to care and vaccination are common across populations in lower-middle income and high income countries [27–29], refugee populations face an additional barrier of being a mobile population. In general, loss to follow up among migrants is worsened by requiring multiple visits to healthcare facilities, involving different healthcare specialists, and lacking appropriate cultural adaptions [30]. Additionally, tracking the status of vaccination, testing, and treatment may be difficult among these transitory populations. One study mentioned that screening interventions are only effective when supported by appropriate follow-up and linkage to care [31], while another study debated the usefulness of HBV and HCV screening, given the expensive treatment and long-term management that it requires in a highly mobile population [32]. Potential methods to explore in supporting continuity of care are retaining refugees’ health and contact information in the healthcare system and building easier pathways for refugees to enter a healthcare system. An electronic health record strategy was also recommended by a previous systematic review of screening barriers for migrants in the European Union [33]. Point of care and/or reflex testing are strategies that can be expanded across interventions to promote screening acceptance and expedite linkage to care [34].

Future implementation of harm reduction models should also be considered. No harm reduction interventions were found in our review. Previous research suggests that injection drug use could be a potential risk factor among refugee populations [35–39]; however, more research is needed on this topic.

Cost-effectiveness studies were limited to HBV, and results were inconclusive on the cost-effectiveness of different combination packages of hepatitis interventions. These differences were possibly due to varying intervention contexts, such as country and setting. Overall, the Australian study and two studies from the United States demonstrated the cost-effectiveness of HBV treatment for refugees [18, 21, 22]. Vaccination was found to be cost-effective by three studies: one from the United States [23], and two studies from refugee camps in South Sudan and the African region [24, 25]. Additional economic analyses are needed to inform program planning.

Political climate and limited humanitarian capacity may pose a challenge to hepatitis service delivery for refugees. Discourse around issues concerning refugees and migrants can be tense for some governments. In Europe and the United States, for example, policies towards migrants tend to be volatile, election-dependent, and poorly coordinated with each other [40]. Furthermore, humanitarian responses are often limited to ensuring migrants’ survival due to financial and time constraints 94]. To support the improvement of hepatitis services for refugees, it will also be necessary to raise general awareness about refugee health and address the stigma around refugees.

The first strength of this review was filling a major literature gap by summarizing key characteristics of hepatitis-related interventions for refugee populations globally to date. A second strength of this review was its comprehensive nature. Three databases were searched and 328 unique records screened for inclusion. Data on key logistics of program implementation, such as location, recruitment, outreach, staffing, and funding sources, as well as program outcomes and accessibility features were extracted from the included studies. Key results from the included prevalence and economic modelling studies were also extracted.

This scoping review had at least three limitations to be noted. First, not all studies provided the same amount of information on intervention logistics, so there was a substantial amount of missing data for details about equipment, recruitment, cost, and other aspects. For example, studies were often not explicit in describing their screening strategy, including whether point-of-care and/or reflex testing was utilized. Second, program outcomes and impact information were not available for most studies, so intervention characteristics could not be systematically evaluated. Third, determining the definition of refugees for study inclusion criteria was challenging. Refugee status depends on the country, and many articles used the broad term of “migrants” to describe their study population. For this scoping review, if an article explicitly mentioned that there were refugees, asylum seekers, or internally displaced persons in their study, then the study met inclusion criteria. This categorization could have left out articles whose study population did include refugees but did not explicitly reference them in their description of their study population.

Moving forward, additional studies on HBV and HCV prevention, testing, and treatment interventions for refugee populations are needed in low- and- middle-income countries. To support program evaluation and replication, it is necessary for interventions to specify implementation details such as testing equipment, cost, funding sources, and program outcomes in more depth. Other models are needed for ensuring continuity of care for refugees who need linkage to care, treatment, and vaccinations. Governments should also look to include refugees in the national hepatitis scheme and provide funding for hepatitis prevention and management services to refugees.

## Conclusions

This is the first scoping review to characterize the types of hepatitis prevention, screening, and treatment interventions serving refugee populations globally. Published experiences of HBV and HCV services for refugee populations remain limited. Only about half of all published experiences including in this scoping review described implementation studies. Most evidence is from high-income countries and there is a lack of consistent dissemination of funding sources, recruitment strategies, and implementation outcomes. Across available studies, community stakeholder participation, bilingual services, and governmental support were noted as key factors to delivering hepatitis services to these diverse populations. Challenges remain in supporting continuity of care for refugee populations. In order to achieve hepatitis elimination globally, best practices must be identified and shared to expand access to hepatitis services for refugee populations.

### Supplementary Information

**Additional file 1:** **Supplementary Table 1.** Cochrane, Embase, and PubMed search syntax.

**Additional file 2**: **Supplementary Table 2.** Individual study characteristics (*n*=69).

**Additional file 3**: **Supplementary Table 3.** Prevalence study setting (*n*=23).

**Additional file 4**: **Supplementary Table 4.** HBV and HCV prevalence (*n*=23).

**Additional file 5**: **Supplementary Table 5.** Testing approaches: Point-of-care testing and reflex testing (*n*=30).

**Additional file 6**: **Supplementary Table 6.** Intervention outcomes (*n*=27).

**Additional file 7**: **Supplementary Table 7.** Funding sources (*n*=38).

**Additional file 8:** **Supplementary Table 8.** Summary of economic modelling studies (*n*=8).

## Data Availability

All data generated or analyzed during this study are included in this published article and its supplementary information files.

## Abbreviations

Anti-HBc: Total antibody to Hepatitis B core antigen
anti-HCV: Hepatitis C antibody
HBV: Hepatitis B
HBsAg: Hepatitis B surface antigen
HBsAb: Hepatitis B surface antibody
HBV DNA: Hepatitis B deoxyribonucleic acid
HCV: Hepatitis C
HCV RNA: Hepatitis C ribonucleic acid
WHO: World Health Organization
