# Supplementary material for: Prevention, testing, and treatment interventions for hepatitis B and C in refugee populations: results of a scoping review
Source: BMC Infect Dis. 2023 Dec 9;23:866. doi: 10.1186/s12879-023-08861-1 (PMC10709891; doi:10.1186/s12879-023-08861-1)
Supplement: Supplementary file 1 — Additional file 1: Supplementary Table 1. Cochrane, Embase, and PubMed search syntax. [file 12879_2023_8861_MOESM1_ESM.docx]

Supplementary Table 1. Cochrane, Embase, and PubMed search syntax

| Cochrane | | |
| --- | --- | --- |
|  | Hepatitis | "hepatitis c":ti,ab OR "hepatitis b":ti,ab OR hcv:ti,ab OR hbv:ti,ab OR chc:ti,ab OR chb:ti,ab OR [mh "hepatitis c"] OR [mh "hepacivirus"] OR [mh "hepatitis b"] |
|  | Population | [mh refugees] OR “internally displaced”:ab,ti OR refugee:ab,ti |
| Embase | | |
|  | Hepatitis | hepatitis c':ti,ab OR 'hepatitis b':ti,ab OR hcv:ti,ab OR hbv:ti,ab OR chc:ti,ab OR chb:ti,ab OR 'hepatitis c'/exp OR 'hepatitis c' OR 'hepacivirus'/exp OR hepacivirus OR 'hepatitis b'/exp OR 'hepatitis b' |
|  | Prevention, testing, treatment | intervention OR program* OR educat* OR support* OR integrat* OR campaign* OR outreach OR counsel* OR engage* OR train* OR teach* OR diagnos* OR treat* OR link* OR 'hepatitis b vaccin*' OR 'opiate substitution' OR 'opioid substitution' OR 'birth dose' OR 'needle syringe' OR 'needle exchange' OR 'direct acting antiviral' OR 'directly observed' OR 'directly observed therapy'/exp OR 'direct observed' OR 'harm reduction'/exp OR screen* OR test* |
|  | Population | refugees'/exp OR 'internally displaced':ti,ab OR refugee:ti,ab |
| Pubmed | | |
|  | Hepatitis | "hepatitis c"[TIAB] OR "hepatitis b"[TIAB] OR hcv[TIAB] OR hbv[TIAB] OR chc[TIAB] OR chb[TIAB] OR "hepatitis c"[Mesh] OR hepacivirus[Mesh] OR "hepatitis b"[Mesh] |
|  | Prevention, testing, treatment | intervention OR program* OR educat* OR support* OR integrat* OR campaign* OR outreach OR counsel* OR engage* OR train* OR teach* OR diagnos* OR treat* OR link* OR "hepatitis b vaccin*" OR "opiate substitution" OR "opioid substitution" OR "birth dose" OR "needle syringe" OR "needle exchange" OR "direct acting antiviral" OR "directly observed" OR "directly observed therapy"[Mesh] OR "direct observed" OR "harm reduction"[Mesh] OR screen* OR test* |
|  | Population | refugees[Mesh] OR “internally displaced”[TIAB] OR refugee[TIAB] |
