# Supplementary material for: Prevention, testing, and treatment interventions for hepatitis B and C in refugee populations: results of a scoping review
Source: BMC Infect Dis. 2023 Dec 9;23:866. doi: 10.1186/s12879-023-08861-1 (PMC10709891; doi:10.1186/s12879-023-08861-1)
Supplement: Supplementary file 2 — Additional file 2: Supplementary Table 2. Individual study characteristics (n=69). [file 12879_2023_8861_MOESM2_ESM.docx]

Supplementary Table 2. Individual study characteristics (n=69)

| **Author, Publication Year** | **Country or region** | **Disease targeted: HBV, HCV, or integrated** | **Purpose: economic modelling, implementation, or prevalence** | **Intervention** | **Intervention setting** | **Reach** |
| --- | --- | --- | --- | --- | --- | --- |
| Ackermann, 2018 | Germany | Integrated (HBV included) | Prevalence | Testing | No data | 94843 serum samples |
| Adachi, 2013 | United States | Integrated (HBV included) | Economic modeling | Vaccination | Clinic | 549-811 patients |
| Ali, 2022 | Bangladesh | Both | Prevalence | Testing, Referral to care | Refugee camp | 300 pregnant women in Oct 2017, 2000 individuals > 7 years old in Feb 2019 |
| Andersen, 2020 | Denmark | Integrated (HBV included) | Prevalence | Testing, Referral to care | Hospital | 160 adult refugees |
| Angeletti, 2016 | Italy | Integrated (HBV and HCV included) | Prevalence | Testing | Clinic | 48 refugees |
| Aşgin, 2019 | Turkey | Both | Prevalence | Testing | Hospital | 809 patients |
| Ash, 2018 | Australia | HBV | Implementation | Education, Testing, Referral to care, Vaccination | Primary health center | 420 refugees |
| Ayele, 2020 | Ethiopia | Both | Prevalence | Testing | Refugee camp | 473 refugees |
| Banks, 2016 | Thailand | Integrated (HBV included) | Implementation | Education, Testing | 3 antenatal clinics along the Thailand-Myanmar border (2 migrant sites and 1 refugee site) | 6158 pregnant women |
| Bergevin, 2021 | France | Integrated (HBV included) | Implementation | Testing, Referral to care, Treatment, Vaccination | Hospital | 128 patients attended consultation, 107 were included in study (rest had missing data) |
| Berman, 2017 | United States | Integrated (HBV included) | Implementation | Vaccination | 12 private clinic sites and community health centers | 792 asylum seekers |
| Bertelsen, 2018 | United States | Integrated (HBV included) | Implementation | Testing, Referral to care, Treatment, Vaccination | Clinic | 239 clients accepted into program; 210 had an appointment with a PCP in the medical clinic and were included in data analysis |
| Bierhoff, 2020 | Thailand | HBV | Implementation | Education, Testing, Referral to care, Treatment, Vaccination | 1 of 3 antenatal clinic sites along Thailand-Myanmar border | 171 pregnant women (16-49 years old) |
| Bozorgmehr, 2017 | Germany | Integrated (HBV included) | Economic modeling | Testing | No data | 324796 asylum seekers >= 16 years old |
| Buonfrate, 2018 | Italy | Integrated (HBV and HCV included) | Implementation | Testing, Referral to care | 14 refugee shelters in Verona province, northern Italy | 481 asylum seekers |
| Chahal, 2019 | United States | HBV | Economic modeling | Testing, Treatment, Vaccination | Not applicable | Not applicable |
| Chandrasekar, 2015 | United States | HBV | Implementation | Education, Testing, Referral to care | 3 clinical and 10 nonclinical sites | 758 individuals |
| Chandrasekar, 2016 | United States | HBV | Implementation | Education, Testing, Referral to care, Vaccination | Several clinical and non-clinical sites | approximately 3000 immigrants |
| Chernet, 2018 | Switzerland | Integrated (HBV and HCV included) | Prevalence | Testing, Referral to care | Refugee center | 107 asylum seekers |
| Colucci, 2022 | Italy | Integrated (HBV and HCV included) | Implementation | Education, Testing, Referral to care | No data | 362 patients |
| Coppola, 2015 | Italy | Integrated (HBV and HCV included) | Implementation | Education, Testing, Referral to care | 4 primary care clinical centers and 2 tertiary units in infectious diseases | 882 patients |
| Coppola, 2017 | Italy | Integrated (HBV and HCV included) | Implementation | Education, Testing, Referral to care, Treatment | 6 clinical centers | 1212 people |
| Cortier, 2022 | France | Integrated (HBV and HCV included) | Implementation | Testing | Centre de Premier Accueil de La Chapelle (accomodation for male asylum seekers) | 2047 migrants |
| Cuomo, 2019 | Italy | Integrated (HBV and HCV included) | Prevalence | Testing | Clinic of Infectious Diseases in Modena | 304 migrants and asylum seekers |
| Del Pinto, 2018 | Italy | Integrated (HBV and HCV included) | Prevalence | Testing, Referral to care | Clinical center | 93 adult refugees |
| Donisi, 2020 | Italy | Integrated (HBV and HCV included) | Implementation | Education, Testing, Referral to care, Vaccination | Hospital | 315 asylum seekers |
| Esmaili, 2021 | United States | Integrated (HBV included) | Prevalence | Testing, Referral to care | Clinic at local health department | 327 pediatric refugees |
| Fiore, 2021 | Italy | Integrated (HBV and HCV included) | Implementation | Testing, Referral to care, Treatment | Immigration center | 81 patients |
| Gargano, 2016 | South Sudan | Integrated (HBV included) | Economic modeling | Vaccination | Yida camp during a humanitarian emergency | Not applicable |
| Hannula, 2021 | Norway | HCV | Implementation | Education, Testing, Referral to care | Local Trondheim refugee center, local prison in Trondheim, OST^1^ clinic, PWUD^2^ housing, Trondheim NEP^3^, outpatient clinics, PWUD^2^ day centers | 381 participants (52 immigrants) |
| Hargreaves, 2020 | United Kingdom | Integrated (HBV and HCV included) | Implementation | Testing, Referral to care | Emergency Department | 96 migrants (13 refugees/asylum seekers) |
| Jablonka, 2017 | Germany | Integrated (HBV and HCV included) | Prevalence | Testing, Vaccination | Single reception center | 618 refugees |
| Janda, 2020 | Germany | Integrated (HBV and HCV included) | Implementation | Education, Testing, Referral to care, Vaccination | Single private pediatric practice | 890 participants |
| Jazwa, 2015 | United States | HBV | Economic modeling | Testing, Referral to care, Treatment, Vaccination | Not applicable | 26548 refugees |
| Johnston, 2012 | Australia | Integrated (HBV and HCV included) | Implementation | Testing, Referral to care | Clinic | 187 refugees |
| Kamali, 2021 | Rwanda | Both | Prevalence | Education, Testing, Referral to care, Treatment | Refugee camp | 26498 individuals |
| Kazmi, 2022 | Pakistan | Both | Prevalence | Testing | Refugee camp | 1225 refugees |
| Khan, 2018 | Pakistan | Both | Prevalence | Testing | No data | 1000 IDPs^4^ |
| Khan, 2011 | Pakistan | HBV | Prevalence | Testing | Medical camps for IDPs^4^ | 950 IDPs^4^ |
| Kowo, 2021 | Cameroon | Integrated (HBV and HCV included) | Prevalence | Education, Testing | Mbile Refugee camp | 970 refugees |
| Lee, 2015 | United States | Integrated (HBV included) | Implementation | Education | Metta Health Center | Not applicable |
| Linde, 2016 | United States | HBV | Implementation | Education, Referral to care | Clinics | 174 refugees |
| MaaBen, 2017 | Germany | Integrated (HBV and HCV included) | Implementation | Testing | Department of Tropical Medicine at the Bernhard Nocht Institute | 190 refugees |
| Mazzitelli, 2021 | Italy | HBV | Implementation | Testing, Referral to care, Treatment, Vaccination | Outpatient clinic | 330 asylum seekers and refugees |
| Mellou, 2019 | Greece | Integrated (HBV included) | Implementation | Education, Vaccination | 2 community healthcare centers in each of the 7 health regions in Greece to reach refugees in the community; RICs^5^; camps | 8474 refugee children and unaccompanied minors |
| Mitchell, 2021 | United States | Integrated (HBV included) | Implementation | Education, Testing, Referral to care, Treatment, Vaccination | Clinical sites, mobile medical teams, outside medical facilities in greater than 80 countries on 5 continents | 320000 refugees |
| Mitchell, 2018 | Thailand | Integrated (HBV included) | Implementation | Education, Testing, Referral to care, Treatment, Vaccination | Refugee camp | 2004 refugees |
| Nyirahabihirwe, 2022 | Rwanda | Both | Implementation | Education, Testing, Referral to care, Treatment | Refugee camp | 26498 refugees |
| Odimayo, 2020 | Nigeria | Both | Prevalence | Education, Testing, Referral to care, Vaccination | Refugee camp at Christian Resource Center | 346 IDPs^4^ |
| Pavlopoulou, 2017 | Greece | Integrated (HBV and HCV included) | Prevalence | Testing | Outpatient clinic of a tertiary children's hospital | 300 migrant children |
| Paxton, 2012 | Australia | Integrated (HBV and HCV included) | Prevalence | Testing | No data | 1136 Karen refugees |
| Payton, 2021 | United States | HBV | Implementation | Testing, Referral to care | 4 primary care clinics, 1 public health department clinic | 12934 refugees |
| Raines-Milenkov, 2021 | United States | Integrated (HBV included) | Implementation | Education, Testing, Referral to care | Clinical and community settings | 1069 refugees |
| Rauf, 2011 | Pakistan | Both | Prevalence | Testing | Refugee camp | No data |
| Reardon, 2019 | African region | HBV | Economic modeling | Vaccination | Refugee camp | No data |
| Rossi, 2013 | Canada | HBV | Economic modeling | Testing, Referral to care, Treatment, Vaccination | No data | Not applicable |
| Russo, 2016 | Italy | Integrated (HBV and HCV included) | Prevalence | Testing, Referral to care | Reception center | 792 asylum seekers |
| Sagnelli, 2018 | Italy | HCV | Implementation | Education, Testing, Referral to care, Treatment | 5 first level clinic centers, 3 tertiary clinics of infectious diseases | 2032 immigrants |
| Schulz, 2014 | Australia | Integrated (HBV and HCV included) | Implementation | Education, Treatment | Telehealth clinic | 120 undocumented immigrants and low-income refugees |
| Serre-Delcor, 2018 | Spain | Integrated (HBV and HCV included) | Prevalence | Education, Testing, Vaccination | Clinic | 303 asylum seekers |
| Sievert, 2018 | Australia | HBV | Implementation | Education | Monash Health Liver and Refugee clinic | 25 Rohingyan refugees and 10 Afghan refugees |
| Stevens, 2016 | India | HBV | Prevalence | Testing | No data | 2769 Tibetan refugees |
| Subramaniam, 2011 | Australia | HBV | Economic modeling | Testing, Treatment | Hospital | No data |
| Tittala, 2018 | Finland | Integrated (HBV included) | Implementation | Testing | Reception center | 37614 asylum seekers |
| Tocco-Tussardi, 2021 | Italy | Integrated (HBV included) | Implementation | Testing | Asylum Seekers Centers of Verona | 715 refugees diagnosed with latent tuberculosis infection, of whom 593 refugees were offered screening (as they were eligible for treatment) |
| Vita, 2019 | Italy | Integrated (HBV included) | Implementation | Vaccination | Internal healthcare facility at an accommodation center for asylum seekers | 3941 migrants |
| Walters, 2016 | United States | HBV | Implementation | Education, Testing, Referral to care, Treatment | Clinic, apartment complexes, onsite clinics at refugee and immigrant organizations | 2087 refugees and foreign born individuals |
| Watanabe-Galloway, 2018 | United States | Integrated (HCV included) | Implementation | Education | Somali Community Service Center | 52 refugee women attended the educational session; 5 people attended the focus group after the session |
| Young, 2020 | United States | HBV | Implementation | Education, Testing, Referral to care, Treatment | Clinics in 3 different cities (Denver - clinic A, St. Paul - clinic B, Philadelphia - clinic C) | Clinic A: 204 HBV+ refugees ; Clinic B: 310 HBV+ refugees; Clinic C: 53 HBV+ refugees |

^1^Opioid substitution therapy

^2^Persons who use drugs

^3^Needle exchange program

^4^Internally displaced persons

^5^Reception and identification center
