# Supplementary material for: Prevention, testing, and treatment interventions for hepatitis B and C in refugee populations: results of a scoping review
Source: BMC Infect Dis. 2023 Dec 9;23:866. doi: 10.1186/s12879-023-08861-1 (PMC10709891; doi:10.1186/s12879-023-08861-1)
Supplement: Supplementary file 3 — Additional file 3: Supplementary Table 3. Prevalence study setting (n=23). [file 12879_2023_8861_MOESM3_ESM.docx]

Supplementary Table 3. Prevalence study setting (n=23)

| 1. **Prevalence study location** | |
| --- | --- |
| Country | Frequency (%) |
| Australia | 1 (4) |
| Bangladesh | 1 (4) |
| Cameroon | 1 (4) |
| Denmark | 1 (4) |
| Ethiopia | 1 (4) |
| Germany | 2 (9) |
| Greece | 1 (4) |
| India | 1 (4) |
| Italy | 4 (17) |
| Nigeria | 1 (4) |
| Pakistan | 4 (17) |
| Rwanda | 1 (4) |
| Spain | 1 (4) |
| Switzerland | 1 (4) |
| Turkey | 1 (4) |
| US | 1 (4) |
| 1. **Prevalence study setting** | |
| Setting | Frequency (%) |
| Clinic or hospital | 8 (35) |
| Medical camps for IDPs^1^ | 1 (4) |
| Reception center | 2 (9) |
| Refugee camp | 7 (30) |
| Refugee center | 1 (4) |
| No data | 4 (17) |

^1^Internally displaced persons
