# Supplementary material for: Prevention, testing, and treatment interventions for hepatitis B and C in refugee populations: results of a scoping review
Source: BMC Infect Dis. 2023 Dec 9;23:866. doi: 10.1186/s12879-023-08861-1 (PMC10709891; doi:10.1186/s12879-023-08861-1)
Supplement: Supplementary file 4 — Additional file 4: Supplementary Table 4. HBV and HCV prevalence (n=23). [file 12879_2023_8861_MOESM4_ESM.docx]

Supplementary Table 4. HBV and HCV prevalence (n=23)

| **Author** | **HBV, HCV, or both** | **Reach** | **HBsAg prevalence** | **Anti-HCV prevalence** | **HBV DNA if provided** | **HCV RNA (viral load) if provided** | **Other HBV markers if provided** |
| --- | --- | --- | --- | --- | --- | --- | --- |
| Ackermann et al | HBV | 94843 serum samples | 3% | No data | No data | No data | No data |
| Ali et al | Both | 2000 refugees | 4% | 11% | No data | No data | No data |
| Andersen et al | Both | 160 adult refugees received the GHA^1^ | 1% | 1% | No data | No data | No data |
| Angeletti et al | Both | 48 refugees | 0% | 0% | No data | No data | No data |
| Aşgin and Satilmiş | Both | 809 patients | 2% | 1% | No data | No data | No data |
| Ayele et al | Both | 473 refugees | 7% | 2% | No data | No data | No data |
| Chernet et al | Both | 107 asylum seekers | 2% | 0% | No data | No data | No data |
| Cuomo et al | Both | 304 migrants and asylum seekers | 12% | 3% | No data | No data | No data |
| Del Pinto et al | Both | 93 adult refugees | 23% | 20% | No data | No data | No data |
| Esmaili et al | HBV | 327 pediatric refugees | No data | No data | No data | No data | 1% - positive for HBV infection based on HBsAg and anti-HBc |
| Jablonka et al | Both | 618 refugees | 3% | 1% | No data | No data | No data |
| Kamali et al | Both | 26,498 individuals screened | 4% | 1% | No data | No data | No data |
| Kazmi et al | Both | 1225 refugees | 7% | 18% | No data | No data | No data |
| A. Khan and J. Qazi | Both | 1000 IDPs^2^ | 5% | 5% | 4% - positive for HBV DNA (viral load) | 4% | No data |
| Khan, F et al | HBV | 950 IDPs^2^ | 6% were HBsAg/HBeAg positive, 25% were Anti-HBs/Anti-HBe positive, and 4% were HBsAg positive only | No data | 21% - positive for HBV DNA (viral load) | No data | No data |
| Kowo et al | Both | 970 refugees | 8% | 3% | No data | No data | No data |
| Odimayo et al | Both | 346 IDPs^2^ | 16% | 1% | No data | No data | No data |
| Pavlopoulou et al | Both | 300 children | 0% | 0.6% | No data | No data | No data |
| Paxton et al | Both | 1136 Karen refugees | 10% | 2% | No data | No data | 9% - isolated hepatitis B core antibody |
| Rauf et al | Both | 590 IDPs^2^ | 0.9% | 9% | No data | No data | No data |
| Russo et al | Both | 792 asylum seekers | 10%* | 3%* | No data | No data | 10% - positive for HBV infection confirmed by HBsAg, HBs-Ab, and HBV DNA |
| Serre-Delcor N, et al. | Both | 303 asylum seekers | 4% | 2% | No data | No data | No data |
| Stevens et al | HBV | 2769 Tibetan refugees | 9% | No data | No data | No data | No data |

^1^General health assessment

^2^Internally displaced persons

*these percents were calculated based on data that was reported in the article
