# Supplementary material for: Prevention, testing, and treatment interventions for hepatitis B and C in refugee populations: results of a scoping review
Source: BMC Infect Dis. 2023 Dec 9;23:866. doi: 10.1186/s12879-023-08861-1 (PMC10709891; doi:10.1186/s12879-023-08861-1)
Supplement: Supplementary file 5 — Additional file 5: Supplementary Table 5. Testing approaches: Point-of-care testing and reflex testing (n=30). [file 12879_2023_8861_MOESM5_ESM.docx]

Supplementary Table 5. Testing approaches: Point-of-care testing and reflex testing (n=30)

| 1. **Intervention included a testing component** | |  |
| --- | --- | --- |
|  | Yes | 30 (79) |
|  | No | 8 (21) |
| 1. **Point of care testing** | | **Frequency (%)** |
|  | Yes | 7 (23) |
|  | No | 15 (50) |
|  | No data | 8 (27) |
| 1. **Reflex testing** | | **Frequency (%)** |
|  | Yes | 12 (40) |
|  | No | 14 (47) |
|  | No data | 4 (13) |
