# Supplementary material for: Prevention, testing, and treatment interventions for hepatitis B and C in refugee populations: results of a scoping review
Source: BMC Infect Dis. 2023 Dec 9;23:866. doi: 10.1186/s12879-023-08861-1 (PMC10709891; doi:10.1186/s12879-023-08861-1)
Supplement: Supplementary file 6 — Additional file 6: Supplementary Table 6. Intervention outcomes (n=27). [file 12879_2023_8861_MOESM6_ESM.docx]

Supplementary Table 6. Intervention outcomes (n=27)

| **Author, Publication year** | **Disease targeted: HBV, HCV, or integrated** | **Intervention** | **Outcome** |
| --- | --- | --- | --- |
| 1. Educational outcomes | | | |
| Sievert, 2018 | HBV | Education | There were 10 participants in the Rohingyan forum and 25 participants in the Afghan forum. Participants in the Afghan and Rohingya community forums showed significant increase in HBV knowledge improvement from pre- and post-forum surveys, including improved knowledge on correct HBV transmission routes. However, incorrect perceptions that HBV could be cured persisted in the Rohingyan group. One Rohingyan patient visited his GP to initiate follow‐up for his previous diagnosis and unmanaged HBV after the forum. The HBV focused radio program was downloaded 5000 times per month and streamed 2000 times per month nationally. |
| 1. Screening uptake | | | |
| Bertelsen, 2018 | Integrated (HBV included) | Testing, Referral to care, Treatment, Vaccination | 96% (n=202*/210) HBV screening coverage |
| Buonfrate, 2018 | Integrated (HBV and HCV included) | Testing, Referral to care | 95%* (n=457/481) HBsAg screening coverage, 25%* (n=118/481) HCV screening coverage |
| Chandrasekar, 2016 | HBV | Education, Testing, Referral to care, Vaccination | 45% (n=445/1000) HBV screening coverage |
| Coppola, 2015 | Integrated (HBV and HCV included) | Education, Testing, Referral to care | 95%* (n=882/926) screening coverage |
| Cortier, 2022 | Integrated (HBV and HCV included) | Testing | 26% (n=49/188) screening coverage |
| Nyirahabihirwe, 2022 | Both | Education, Testing, Referral to care, Treatment | 78% (n=26498/34000) screening coverage |
| Raines-Milenkov, 2021 | Integrated (HBV included) | Education, Testing, Referral to care | 38% (n=409/1069) HBV screening coverage |
| Tocco-Tussardi, 2021 | Integrated (HBV included) | Testing | 36% (n=211/593) of refugees accepted screening for HBV |
| Tittala, 2018 | Integrated (HBV included) | Testing | 61% screening coverage for HBV, HIV, or syphilis. |
| 1. Linkage to care | | | |
| Chandrasekar, 2015 | HBV | Education, Testing, Referral to care | In the clinical setting, 56% (9/16) HBsAg+ individuals referred to follow up care, 19%* (3/16) refused further care, and 25%* (4/16) could not be contacted. In the non-clinical settings: 77% (30/39) HBsAg+ individuals referred to follow-up medical care and 23%* (9/39) could not be contacted |
| Linde, 2016 | HBV | Education, Referral to care | Before intervention (1 year): 64% (56/87) HBsAg+ individuals received follow-up care, 14% (12/87) did not receive follow-up care, 22% (19/87) could not be located After intervention (1 year): 93% (162/174) HBsAg+ received follow-up care, 4% (7/174) did not receive follow up care, and 3% (5/174) could not be located |
| Payton, 2021 | HBV | Testing, Referral to care | Of the 682 HBsAg+ individuals: 20% had HBV specialist referrals within 30 days and 36% were seen within 6 months |
| Walters, 2016 | HBV | Education, Testing, Referral to care, Treatment | 94% (72/77) of HBsAg+ individuals were successfully linked to care; 94% (68/72) attended their first follow-up visit. Patients were seen for follow-up within 90 days of their positive test |
| Young, 2020 | HBV | Education, Testing, Referral to care, Treatment | Clinic A: 29% (60/204) of HBsAg+ patients linked to care, 12% (24/204) retained in care, 84% (172/ 204) not receiving optimal care, and 1 person died. Clinic B: 21% (29/137) of HBsAg+ patients retained in care and 79% (108/137) not receiving optimal care. Clinic C: 53% (28/53) of HBsAg+ patients linked to care, 11% (6/53) retained in care, 89% (47/53) not receiving optimal care |
| 1. Screening uptake and linkage to care | | | |
| Fiore, 2021 | Integrated (HBV and HCV included) | Testing, Referral to care, Treatment | 77% (62/81) HCV screening coverage. 1 person diagnosed with HCV and dropped out before treatment. 72% (58/81) HBV screening coverage and all 3 patients who needed HBV treatment dropped out without receiving treatment |
| 1. Screening uptake and treatment outcomes | | | |
| Sagnelli, 2018 | HCV | Education, Testing, Referral to care, Treatment | 85% (1727/2032) screening coverage. 2/2 patients who received interferon-free treatment were cured after 6 months. 4/6 patients who received interferon-based treatment were cured. |
| 1. Screening uptake, linkage to care, and treatment outcomes | | | |
| Coppola, 2017 | Integrated (HBV and HCV included) | Education, Testing, Referral to care, Treatment | 91% (1212/1331) screening coverage 78% (90/116) of HBsAg+ individuals completed their diagnostic itinerary at a tertiary infectious disease unit. 1/5 patients treated with peginterferon α-2a for 12-24 months obtained a favorable response (HBV treatment) 9/9 patients treated with nucleoside or nucleotide analogues were serum HBV DNA negative within the 48th week of treatment |
| 1. Vaccine uptake | | | |
| Berman, 2017 | Integrated (HBV included) | Vaccination | n=2269 refugees (adults and children) At least 1 dose: 91% 2 doses: 63% 3 doses: 10% |
| Mellou, 2019 | Integrated (HBV included) | Education, Vaccination | n=1509 children First dose: 49% (733) Second dose: 25% (372) |
| Mitchell, 2021 | Integrated (HBV included) | Education, Testing, Referral to care, Treatment, Vaccination | n=28478 refugees (adults and children) First dose: 87% Second dose: 77% |
| Vita, 2019 | Integrated (HBV included) | Vaccination | n=118 minors First dose: 51%* (60) Second dose: 34%* (40) Third dose: 7%* (8) n=3823* adults** First dose: 0.5%* (18) Second dose: 0.2%* (7)* Third dose 0.03%* (1) |
| Contact tracing and vaccine uptake |  |  |  |
| 1. Contact tracing and vaccine uptake | | | |
| Ash, 2018 | HBV | Education, Testing, Referral to care, Vaccination | Of the 420 contacts, 90% were successfully traced and 6% (27) of susceptible contacts were immunized (adults and children) |
| 1. Screening uptake, linkage to care rates, vaccine uptake | | | |
| Mazzitelli, 2021 | HBV | Testing, Referral to care, Treatment, Vaccination | 88%* screening coverage (203*/231). After 6 months, 22% (5/23) of HBsAg+ patients were lost to follow up or transferred from their hosting centers. After 12 months, 43% (10/23) of patients were retained in care. At 6 months follow up, 57% (116/204) adults and children had been vaccinated, 18% (35/304) had the vaccination course ongoing, and 17% (35/204) had not started vaccination course. At 12 months, 92% (131/142 migrants still residing at the centers) vaccination coverage, 1% (2/142) had the vaccination course ongoing, and 6% (9/142) had not started vaccination course. |
| Mitchell, 2018 | Integrated (HBV included) | Education, Testing, Referral to care, Treatment, Vaccination | 57% (2004/3419) screening coverage. 191 tested HBsAg+ and received evaluation and counseling. 99% (1564/1576 eligible adults and children) received at least 1 dose of HBV vaccine |
| 1. Screening uptake, screening timing, linkage to care rates, vaccine uptake | | | |
| Bergevin, 2021 | Integrated (HBV included) | Testing, Referral to care, Treatment, Vaccination | 84%* (90/107) HBV screening coverage. 14%* (1/7) patients treated, 29%* (2/7) patients lost to follow-up, and 57%* (4/7) patients followed up elsewhere. 29% (31/107 children) were vaccinated Median time to COPEMI consultation after migration was 3 months; 40%* (43/107) did not attend the second consultation |
| 1. Miscellaneous outcomes | | | |
| Schulz, 2014 | Integrated (HBV and HCV included) | Education, Treatment | Nearly 500km of travel was avoided per consultation |

*these values were calculated based on data reported

**in adults, vaccinations were given in response to epidemic events or post-exposure prophylaxis
