# Supplementary material for: Prevention, testing, and treatment interventions for hepatitis B and C in refugee populations: results of a scoping review
Source: BMC Infect Dis. 2023 Dec 9;23:866. doi: 10.1186/s12879-023-08861-1 (PMC10709891; doi:10.1186/s12879-023-08861-1)
Supplement: Supplementary file 7 — Additional file 7: Supplementary Table 7. Funding sources (n=38). [file 12879_2023_8861_MOESM7_ESM.docx]

Supplementary Table 7. Funding sources (n=38)

| **Funding Sources** | **Frequency (%)** |
| --- | --- |
| Global non-profit organization | 1 (3) |
| Government | 12 (32) |
| Internal funding | 1 (3) |
| In-kind commodity donations | 3 (8) |
| National health insurance | 8 (21) |
| No data | 18 (47) |
