# Supplementary material for: Prevention, testing, and treatment interventions for hepatitis B and C in refugee populations: results of a scoping review
Source: BMC Infect Dis. 2023 Dec 9;23:866. doi: 10.1186/s12879-023-08861-1 (PMC10709891; doi:10.1186/s12879-023-08861-1)
Supplement: Supplementary file 8 — Additional file 8: Supplementary Table 8. Summary of economic modelling studies (n=8). [file 12879_2023_8861_MOESM8_ESM.docx]

Supplementary Table 8. Summary of economic modelling studies (n=8)

| **Author, publication year** | **Country** | **Intervention strategies** | **Findings** |
| --- | --- | --- | --- |
| Adachi, 2013 | United States | Vaccination | Vaccination had a break-even or slightly positive cost-revenue structure at the DeKalb clinic. Public-sector per-dose price of HBV vaccination was $28.00 for adults and $10.50 for children. |
| Bozorgmehr, 2017 | Germany | Screening | Through statutory insurance, the cost to the healthcare system per identified HBV case was 622.50 EUR^1^. Through private insurance, the cost to the healthcare system per identified HBV case was 1167.50 EUR^1^. Compared to other infectious diseases, HBV had one of the highest cost per identified case with respect to the initial screening test (>600 EUR^1^). |
| Chahal, 2019 | United States | 1) Vaccination only  2) Treatment only  3) Screening, vaccination, and treatment | Vaccination-only and treatment-only strategies had an incremental cost-effectiveness ratios (ICERs) of $6000 to $21000 per quality-adjusted life years (QALY) gained, respectively. Cost per QALY gained vs. no intervention was $17432 for screening, vaccination, and treatment combined. While vaccination-only and treatment-only strategies were cost-effective, an inclusive approach of screening, treatment, and vaccination was incrementally very cost-effective or even cost-saving comparatively. |
| Gargano, 2016 | South Sudan | 1) One dose of combined Hib-containing and PCV vaccines  2) Two doses of combined Hib-containing and PCV vaccines | The cost per disability-adjusted-life-year (DALY) averted for administering combined one- and two-doses was $125 and $209, respectively |
| Jazwa, 2015 | United States | 1) Screen, then vaccinate or initiate management (SVIM)  2) Vaccinate only without screening first (VO) | SVIM was more cost beneficial than VO. Five years after program initiation, SVIM had a positive net benefit of $24-$130 million, depending on domestic post-arrival screening rates in the VO strategy |
| Reardon, 2019 | African region | 1) Universal HBV birth dose + routine immunization (HBV BD + RI)  2) Screen mothers for HBV with rapid diagnostic tests, and provide HBV birth dose for newborns whose mothers tested positive for HBV, in addition to routine immunization (RDT HBV BD + RI) | HBV BD + RI strategy would save 9807 life-years per year, with an ICER of 0.15 USD^2^ per life-year saved in Djibouti camps, 27108 life-years/year with an ICER of 0.11 USD^2^ in Algerian camps, and 18417 life-years/year with an ICER of 0.16 USD^2^ in Mauritanian camps. RDT HBV BD + RI strategy was not cost effective. |
| Rossi, 2013 | Canada | 1) Universal vaccination  2) Screening for prior immunity + vaccination  3) Screening for HBV + treatment  4) Screening for HBV and prior immunity + treatment + vaccination | Screening and treatment for HBV was the most cost-effective ($40880 per additional QALY gained relative to no intervention). Strategies that included HBV vaccination were either prohibitively expensive or dominated by the chronic HBV screening strategy. |
| Subramaniam, 2011 | Australia | Treatment | Without treatment, costs were estimated to increase by 400% from $401460 to $2027078 over 10 years (2010-2020). |

^1^Euros

^2^US dollars

*findings are in US dollars unless specified otherwise
